# Human Male Body Size Predicts Increased Knockout Power, Which Is Accurately Tracked by Conspecific Judgments of Male Dominance

**DOI:** 10.1007/s12110-024-09473-7

**Published:** 2024-06-15

**Authors:** Neil R. Caton, Lachlan M. Brown, Amy A. Z. Zhao, Barnaby J. W. Dixson

**Affiliations:** 1 School of Psychology, University of Queensland, Brisbane, QLD Australia; 2 School of Psychology, The University of the Sunshine Coast, Sunshine Coast, QLD Australia

**Keywords:** Male body size, Hand-to-hand combat, Intrasexual selection, Knockout power, Dominance perception

## Abstract

Humans have undergone a long evolutionary history of violent agonistic exchanges, which would have placed selective pressures on greater body size and the psychophysical systems that detect them. The present work showed that greater body size in humans predicted increased knockout power during combative contests (Study 1a-1b: total *N* = 5,866; Study 2: *N* = 44 openweight fights). In agonistic exchanges reflective of ancestral size asymmetries, heavier combatants were 200% more likely to win against their lighter counterparts because they were 200% more likely to knock them out (Study 2). Human dominance judgments (total *N* = 500 MTurkers) accurately tracked the frequency with which men (*N* = 516) knocked out similar-sized adversaries (Study 3). Humans were able to directly perceive a man’s knockout power because they were attending to cues of a man’s body size. Human dominance judgments—which are important across numerous psychological domains, including attractiveness, leadership, and legal decision-making—accurately predict the likelihood with which a potential mate, ally, or rival can incapacitate their adversaries.

**Supplementary Information:**

The online version contains supplementary material available at 10.1007/s12110-024-09473-7.

Intrasexual selection has shaped the evolution of agonistic displays, fighting styles, and weaponry employed during contest competition (Andersson, 1994; Emlen, 2008). During contests, some morphological traits serve as ‘weapons’ to inflict damage on opponents (Emlen, 2008; McCullough et al., 2016) whereas ornamental features communicate social rank and dominance without necessarily being related to physical formidability (Dixson et al., 2005; Grueter et al., 2015; for more cross-primate evidence of ornamental features, see Aung et al., 2023; but also see Aung et al., 2020; Aung & Puts, 2020; Puts & Aung, 2019; Rendall et al., 2007). However, both improve male resource-holding power (Arnott & Elwood, 2009). One of the most widely used measures of resource-holding power in animal contest research is overall body size (i.e., overall weight itself; Andersson, 1994; Archer, 1988; Arnott & Elwood, 2009; Emlen, 2008; Huntingford, 2013). Human social systems, in particular, are characterized by male-male contest competition (Morris et al., 2020; Puts, 2010), and intrasexual selection is argued to have shaped human body size and composition to increase force output in agonistic exchanges (Lassek & Gaulin, 2009; Puts, 2010; Puts et al., 2023; Sell et al., 2009, 2012).

Male-male contest competition via dyadic combat is an ancient feature of hominin evolution (Carrier & Morgan, 2014; Morgan & Carrier, 2013). Dyadic fighting in the form of hand-to-hand combat has been a strong selective pressure for *Homo sapiens*, which has uniquely shaped men’s facial (Carrier & Morgan, 2014) and fist (Morgan & Carrier, 2013) morphology to buffer against the impact of strikes. This evolutionary legacy is evident in the universal nature of hand-to-hand combat, which is observed among Europeans (Darwin, 1872; Morgan & Carrier, 2013); ingrained as a central feature of Asian martial arts, such as Karate, Kung Fu, and Xing Yi Quan, the Chinese martial art meaning “form and intent fist” (Morgan & Carrier, 2013); discussed in humanity’s earliest written sources (Exodus 21:18; Ezekiel 6:11; Matthew 26:67); recorded in hunter-gatherer populations, such as the Yanomamö of Brazil and Venezuela (Chagnon, 1968); and the cause of 46–67% of fight-associated facial fractures (Carrier & Morgan, 2014).

Given that hand-to-hand combat was a powerful selective force throughout human evolutionary history (Carrier & Morgan, 2014; Morgan & Carrier, 2013), this would have placed selective pressures on the development of morphological and psychological structures which enable the use of long-range weapons (e.g., spears, polearms), projectile weapons (e.g., arrows, darts), defensive armour, coalitionary violence, and the need for community dominance (e.g., reverse dominance hierarchy; see Boehm, 1993). Some of these alternative strategies require strong morphological features (e.g., spears and bows require considerable strength; Grund, 2017; Pontzer et al., 2017) whereas others require distance from the target (e.g., darts, blowpipes), and most human dyadic contests begin when two conspecifics are within each other’s immediate vicinity (Carrier & Morgan, 2014). In situations characterized by coalitionary violence or reverse dominance hierarchy, wherein the community holds dominance over a ruling minority (Boehm, 1993), humans have nonetheless evolved psychological mechanisms for evaluating, choosing, and aligning with larger-bodied individuals as leaders of their coalition or community (Holbrook & Fessler, 2013; Re et al., 2012). This may signify the adaptive advantage of having a larger-bodied leader to ensure their own and others’ survival through effective engagement in coalitionary violence. With the above in mind, there does exist a broader evolutionary context to both hand-to-hand combat and general violence. However, these alternative strategies may actually underscore the adaptive significance of an initial evolution of large morphological features. Large morphological features may have initially evolved, which would have placed selective pressures on the development of alternative strategies (e.g., weaponry, armour) to overcome the evolutionary force of hand-to-hand combat. The emergence and presence of these alternative strategies underscore the evolutionary importance of hand-to-hand combat. Without these alternative strategies, such as weapons or armour, the argument for the evolution of hand-to-hand combat is indeed less plausible.

Sexual selection is also suggested to have shaped human psychological mechanisms that assess and respond to formidability and fighting ability. Those individuals throughout human evolutionary history who were better able to detect men’s resource-holding power from their body size and composition would have avoided costly losses, formed valuable coalitions, and, for women, selected higher-quality mates (Puts, 2010; Sell et al., 2009, 2012). These selective pressures have shaped modern human psychology: preverbal infants use body size to predict the outcome of dominance contests (Thomsen et al., 2011), and people rate men with greater body size as higher in fighting ability and aggressiveness (Caton et al., 2022b, 2023), leadership ability (Holbrook & Fessler, 2013; Re et al., 2012), masculinity (Holzleitner et al., 2014), and persuasiveness (Kniffin et al., 2019). Moreover, body size and muscularity are strong determinants of men’s attractiveness cross-culturally (Anikin et al., 2021; Aung et al., 2021b; Sell et al., 2017), with one study reporting that none of the 160 heterosexual women involved in the study reported statistically significant preferences for men with physically weaker physiques (Sell et al., 2017). However, although contemporary research assumes that men’s body size is positively associated with their fighting success (Anikin et al., 2021; Aung et al., 2021a; Carrier & Morgan, 2014; Caton et al., 2022a, 2022b; Evans et al., 2006; Fessler et al., 2014; Puts, 2010; Zilioli et al., 2014), to our knowledge, research to date has never shown any direct associations between men’s body size and resource-holding power (especially for force output broadly, here examined as knockout power specifically) in agonistic exchanges.^1^

## Body Size and Knockout Power in Human Contest Competition

Mounting evidence from evolutionary biological and psychological research suggests that sexual selection for bodily force output, particular for striking with fists, was a feature of human evolution (for more detail, see Carrier & Morgan, 2014; Caton & Dixson, 2022a; Morgan & Carrier, 2013; Morris et al., 2020). Bodily force output, especially in the form of a knockout punch, can result in severe sensory damage, loss of consciousness, or even death (Fenton et al., 2003; Flynn et al., 2016). Notably, significant brain damage and even death can occur from a single strike to the head (Fenton et al., 2003; Flynn et al., 2016). In biomechanics research, body size is positively associated with the capacity to generate force (Viano et al., 2005; Walilko et al., 2005), which potentially translates to greater knockout power during agonistic exchanges. Only one study (Baird et al., 2010) of which we are aware investigated a relationship between male body size and force output, specifically in the form of knockout power, finding a negative association (see General Discussion for a possible explanation for the discrepancy between this research and the present work, and see note 1).

In addition to obtaining victories via knockouts, fighters employ many fighting manoeuvres during contests, including forcing an opponent to submit and retreat and manoeuvres requiring endurance and sustained aggression (i.e., high frequencies of striking or grappling; Caton & Dixson, 2022a; Caton et al., 2022a; Fenton et al., 2003; Flynn et al., 2016; Lane & Briffa, 2020). Greater body size in nonhuman species is negatively (or nonsignificantly) correlated with these other fighting performance metrics in agonistic exchanges against other conspecifics (Briffa & Lane, 2017; Zamudio et al., 1995). Such agonistic behaviours might not result in an opponent’s immediate death or incapacitation and would therefore be less evolutionarily advantageous than knockout victories, which can immediately result in victory (Fenton et al., 2003; Flynn et al., 2016). Importantly, the benefits associated with increased knockout power may therefore *supersede* the benefits stemming from other fighting manoeuvres. This does not mean that the human body did not evolve to increase the success of fighting manoeuvres (e.g., in a wrestling match with no striking, body size should indeed have an advantage) but rather that the application of force (in the form of a hand-based or weapon-based strike) can result in immediate death. The benefits associated with this immediacy may therefore supersede, and therefore preclude, the evolutionary benefits stemming from other fighting manoeuvres.

## Human Psychology May Track Knockout Power by Attending to Body Size

In addition to driving the evolution of morphological structures employed in contest competition, sexual selection may have shaped human psychological mechanisms to detect, process, and respond appropriately to cues of fighting ability (Archer, 2009; Puts, 2010). Because the costs of fighting for limited resources may be high (e.g., injury, death), sexual selection may have favoured psychological mechanisms that enabled accurate assessment of information regarding the likely costs and benefits of engaging in conflict (Arnott & Elwood, 2009). Human dominance judgments appear accurate at detecting the winners and losers of fights, but Lane and Briffa (2020) note that this accuracy is only observed when participants can compare opponents in a forced-choice paradigm (Little et al., 2015) or only among heavyweight fighters (Třebický et al., 2013). Moreover, research on human dominance judgments has also only examined men’s *overall* ability to win a fight (i.e., total wins divided by total fights; Třebický et al., 2013; Zilioli et al., 2014). Yet, morphological features can act as ornaments that increase the likelihood of individuals entering costly fights in nonhuman species (Dixson et al., 2005; Grueter et al., 2015; McCullough et al., 2016) and humans (Lane & Briffa, 2020; Little et al., 2015; Zilioli et al., 2014). The current study examines whether people can accurately predict the rate at which men have *knocked out* their adversaries. If greater body size was shaped by sexual selection to increase force output in the form of knockout power, then human psychological systems—if evolved to enable accurate information gathering—should also have been shaped by sexual selection to track force output in the form of knockout power from body size. Overall, the present work predicts that (1) human body size should be positively correlated with knockout victories and (2) human perceptions of fighting ability should be positively correlated with fighters’ knockout victories via their body size.

## Study 1

Our first study provided preliminary evidence for the association between force output (in the form of knockout power) and body size in weight-restricted data in mixed-martial-arts (MMA) fights, previously used to examine research questions related to human evolution and contest competition (Caton et al., 2022a, 2022b; Lane & Briffa, 2020; Třebický et al., 2013, 2015; Zilioli et al., 2014). Research with weight-restricted data has previously examined the association between body size and overall fighting success (i.e., total wins divided by total fights) and found a positive association between these variables (Aung et al., 2021a; Palmer-Hague et al., 2018; also see note 1). Because the data are weight-restricted, these past findings confusingly suggest that heavyweight fighters will win more often against heavyweight fighters than lightweight fighters will win against lightweight fighters—yet, every weight-restricted bout results in a winner. To demonstrate an association between body size and resource-holding power in weight-restricted data, a more appropriate analysis would be to demonstrate a positive association between body size and knockout victories. This would indicate that heavier combatants are more likely to knock out their equally-heavy counterparts than lightweight combatants are to knock out their equally-light counterparts. This suggests that heavier combatants possess greater knockout power.

### Study 1a: Method

Data were drawn from a publicly available dataset (Binduvr, 2021) which included fight statistics from 5,151 professional athletes (*M*_age_ = 35.32; *SD* = 6.64) in the Ultimate Fighting Championship (UFC), Bellator Fighting Championship, and One Fighting Championship (Binduvr, 2021). Data were used for the fighters’ weight (referred to in the animal literature as body size; *M*_kg_ = 74.80, *SD* = 16.59) as well as knockout (*M* = 4.05, *SD* = 4.63), submission (*M* = 3.82, *SD* = 4.53), and decision (*M* = 2.91, *SD* = 3.28) wins (which represents the average amount of knockout, submission, and decision victories across all fighters), total fights (*M* = 16.27, *SD* = 12.83), and sex (280 female, 2551 male, and 2320 cases in which these data were not available). Since it would be statistically inappropriate to examine the knockout–body size association only for men—akin to conducting simple effects analyses without observing the interaction—the interaction between sex and body size was used to examine whether the knockout–body size association held specifically for males, females, or did not differ across the sexes.

First, it is not necessary to show that this relationship holds for male-male contests but not female-female contests (Lane & Briffa, 2020) since larger body size should be biomechanically associated with greater force output, regardless of the competitor’s sex. Second, an analysis restricted to males acts as a simple-effects analysis of the larger analysis, in which such an analysis is not restricted to either sex. Before running a simple-effects analysis, researchers must evaluate an interaction term between the two variables of interest to determine whether there is any statistical reason to run a simple-effects analysis (Brambor et al., 2006). This interaction shows whether there is a significant difference between the levels of the moderator (in this case, sex) to justify whether it is statistically appropriate to perform the subsequent simple-effects analysis. It is fundamentally misleading to restrict analyses to the less informative analysis, but it is nonetheless important to note that a nonsignificant interaction term in the present work indicates that the results do not change if we separated the analyses by sex. Finally, human contest competition research literature to date has reported nonsignificant interactions between sex and morphological or fighting performance indicators on the likelihood of victory (Caton & Dixson, 2022a; Lane & Briffa, 2020), which is further substantiated by the results of the present work.

### Study 1b: Method

Fight statistics were collected for all fighters (*M*_age_ = 30.62; *SD* = 4.53) on ufc.com (*N* = 715). Data were collected for fighter’s body size (*M*_kg_ = 74.29; *SD* = 16.65), total fights (*M* = 18.89; *SD* = 10.09), and fighter’s sex (113 females, 602 males). Detailed information on the nature of wins (i.e., by knockout or technical knockout [KO/TKO]: *M* = 5.90; *SD* = 4.24; submission: *M* = 4.39; *SD* = 4.17; decision: *M* = 4.24; *SD* = 3.19) was collected from espn.com. To test whether body size was associated with any other performance metrics, we collected data on fighter’s lifetime attempted (*M* = 661.90, *SD* = 647.23) and landed (*M* = 288.11, *SD* = 275.20) strikes; attempted (*M* = 24.53, *SD* = 31.27) and landed (*M* = 9.34, *SD* = 11.49) takedowns; strikes in standing (*M* = 204.95, *SD* = 238.53), clinch (*M* = 42.72, *SD* = 47.13), and ground (*M* = 47.13, *SD* = 56.33) positions; and strikes to the opponent’s head (*M* = 185.80, *SD* = 178.85), upper body (*M* = 57.20, *SD* = 61.74), and lower body (*M* = 45.72, *SD* = 55.27). Data were also included on average fight duration (in seconds; *M* = 640.80, *SD* = 196.22) and strikes landed per minute (*M* = 3.63, *SD* = 1.52). Detailed definitions for these variables are reported by James et al. (2017). There was no evidence of multicollinearity in any of the following models because all VIF scores were below 10 (Hair et al., 1995).

### Study 1a: Results

Linear regressions were run to examine whether knockout, submission, or decision victories were more frequent among larger-bodied fighters. In line with previous research (Caton et al., 2022a, 2022b; Zilioli et al., 2014), we controlled for the total number of fights. Controlling for total fights, there was a significant positive association between body size and knockout victories, β = 0.15, *t*(5116) = 16.23, *p* < .001, but negative associations between body size and submission, β = −0.06, *t*(5116) = − 6.07, *p* < .001, and decision victories, β = −0.16, *t*(5116) = − 14.75, *p* < .001. We also ran these models controlling for the corresponding avenues to victory, and the same results emerged (see ESM). Because fighters are positioned in weight classes, heavier fighters are more likely to knock out similarly-heavy opponents than lighter fighters are to knock out similarly-light opponents; heavier fighters exhibit greater knockout power. There was no interaction between sex and body size on knockout wins, β = −0.003, *t*(2805) = − 0.02, *p* = .98.

### Study 1b: Results

Similar results were found in our more specific sample of UFC fighters. Controlling for total fights, there was a significant positive association between body size and knockout victories, β = 0.24, *t*(712) = 8.31, *p* < .001, but a negative (nonsignificant) association between body size and submission victories, β = −0.04, *t*(712) = − 1.24, *p* = .22, and a significant negative association between body size and decision victories, β = −0.23, *t*(678) = − 7.63, *p* < .001. We also ran these models controlling for the corresponding avenues to victory, and the same results emerged (see the ESM). These findings further support the argument that larger fighters possess greater knockout power. There was no significant interaction between sex and body size on knockout wins, β = −0.86, *t*(711) = − 1.23, *p* = .22. Further, controlling for total fights and fight duration, there was no significant association between body size and strikes landed, β = −0.02, *t*(640) = − 0.68, *p* = .50; attempted strikes, β = −0.06, *t*(640) = − 1.85, *p* = .07; strikes in a clinch, β = 0.01, *t*(633) = 0.37, *p* = .71, ground position, β = 0.04, *t*(616) = 0.92, *p* = .36, or standing position, β = −0.07, *t*(641) = − 1.83, *p* = .07; and no significant association between body size and strikes landed to an adversary’s upper body, β = −0.05, *t*(639) = − 1.48, *p* = .14, lower body, β = −0.06, *t*(635) = − 1.71, *p* = .09, and head, β = −0.001, *t*(640) = − 0.03, *p* = .98. Controlling for total fights and fight duration, there was no significant association between body size and attempted, β = −0.03, *t*(582) = − 0.64, *p* = .52, or landed, β = 0.01, *t*(487) = 0.17, *p* = .87, takedowns. There was also no significant association between body size and strikes landed per minute, controlling for total fights, β = −0.01, *t*(641) = − 0.35, *p* = .72.

### Study 1: Discussion

Larger body size in humans is associated with greater knockout power. Heavier individuals are more likely to knock out their heavier counterparts than lighter individuals are to knock out their lighter counterparts. These results do not explicitly show that a larger-bodied opponent is more likely to emerge victorious against a lighter-bodied opponent within the same contest. Furthermore, these results do not attest the advantages of larger body size in contests reflective of ancestral size asymmetries. Our next study used openweight contest data—agonistic exchanges with no weight restrictions—which has never before been investigated in the biological, sports, or psychological literature. This allowed us to quantify the extent to which larger body size is associated with fighting success in contests reflective of ancestral size asymmetries, as well as knockout power and fighting performance more specifically.

## Study 2

### Study 2: Method

The Road Fighting Championship (Road FC) contains the largest sample of openweight fights, which are illegal (or in which there are policies against openweight fights) in most countries. This makes the present dataset the only sample of contests without weight restrictions. The present dataset comprised all 44 Road FC openweight contests (6 female fights, 38 male fights) across the organisation’s history up until November 2021 (when the data were collected) for fights where contest-specific weigh-in data was available and which resulted in a victory (i.e., not a tie or no contest ruling). Fight-specific weigh-in data were collected from official websites (tapology.com, sherdog.com, or roadfc.com). An independent research assistant then coded these fights featured on roadfc.com for each fighter’s attempted strikes (*M* = 19.90; *SD* = 17.67), landed strikes (*M* = 36.56; *SD* = 34.71), attempted takedowns (*M* = 0.38; *SD* = 0.93), and landed takedowns (*M* = 0.17; *SD* = 0.51) using definitions provided in James et al. (2017), as well as the method of resolution (i.e., KO/TKO: 32 total; submission: 8 total; or decision: 4 total).

In line with Lane and Briffa (2020), we randomly assigned one fighter to be the focal fighter and their opponent to be the “nonfocal” fighter, which allowed us to avoid any confounding effect of fighting colour on the outcome (average weight discrepancy between fighters: *M*_kg_ = 16.85, *SD* = 13.10). “Fighting colour” refers to the colour associated with the corner station (red or blue) in which a fighter’s team is located (for more information on why fighting colour can represent a confounding variable, see Lane & Briffa, 2020). Both dichotomous (i.e., was the winner heavier than their opponent: 0 = *no*, 1 = *yes*; did the heavier fighter knock out their opponent: 0 = *no*, 1 = *yes*) and continuous (i.e., focal fighter’s weight in kg, controlling for the nonfocal fighter’s weight) measures were used to examine the associations between body size and fighting success and knockout power.

In line with previous animal contest research (Batchelor & Briffa, 2010, 2011; Briffa et al., 2012; Hardy & Briffa, 2013), our dataset was first structured in long format for the execution of maximal multilevel models. These maximal models were then progressively simplified based on Akaike information criterion (AIC) scores, which were assessed in lowest-is-best format at each simplification to ascertain whether the changes enhanced the model fit until a minimum adequate model was reached (Batchelor & Briffa, 2010, 2011; Briffa et al., 2012; Lane & Briffa, 2020). Multilevel logistic models often resulted in a singular fit, uninterpretable parameters, and comparatively high AIC scores; logistic regressions in wide format ultimately proved to be the minimal adequate model.

### Study 2: Results

#### Fighting Success

First, a chi-square goodness-of-fit test was conducted to examine whether the heavier combatant was more likely to emerge victorious against their lighter combatant. Heavier combatants (33 of 44 cases) were 200% more likely to emerge victorious against their lighter counterparts (11 of 44 cases; χ² = 11.00, *p* < .001). Next, we conducted a binomial logistic regression with the focal fighter’s body size (kg) as a continuous predictor of whether the focal (vs. nonfocal) fighter emerged victorious, controlling for the nonfocal fighter’s body size (which allows us to assess the degree to which a focal fighter’s body size *exceeds* their opponent’s body size as a predictor of fighting success). Findings revealed that the greater a fighter’s body size than that of their opponent, the more likely they were to emerge victorious in the agonistic encounter (*OR* = 1.06, *Z* = 2.52, *p* = .01). For every kilogram that a fighter’s body size exceeded that of their opponent, they were 6% more likely to emerge victorious. Consistent with the previous study, there was no interaction between sex and body size on the likelihood of victory (*OR* = 1.07, *Z* = 1.12, *p* = .26).

#### Knockout Power

A chi-square goodness-of-fit test was conducted to examine whether the heavier combatant was more likely to knock out the lighter combatant than the lighter combatant was to knock out the heavier combatant. Heavier combatants (24 of 32 cases) were 200% more likely to win by knockout than their lighter counterparts (8 of 32 cases; χ² = 8.00, *p* = .005). We then conducted a binomial logistic regression with the focal fighter’s body size as a continuous predictor of whether the focal (vs. nonfocal) fighter won by knockout, controlling for the nonfocal fighter’s body size. Findings revealed that, for every kilogram that a fighter’s body size exceeded that of their opponent, they were 5% more likely to knock them unconscious (*OR* = 1.05, *Z* = 2.00, *p* = .045). This highlights the potential role of body size in environments reflective of ancestral size asymmetries: fighters who are 20 kg heavier than their opponent will be 100% more likely to knock out their lighter counterpart. There was no interaction between sex and body size on the likelihood of knockout (see ESM for more detail). It is not statistically appropriate to control for the fighters’ general ability to win (a culmination of knockout, submission, and decision victories) within the specific knockout victory analyses, which would result in null deviance and an overfitted model, especially due to the multicollinearity issues that arise when controlling for highly similar variables.

#### Additional Metrics

Consistent with the previous studies, larger body size afforded no other advantages other than increased knockout power (i.e., landed and attempted strikes or landed and attempted takedowns; see the ESM) in the present work (but refer to the limitations section in the General Discussion for a more detailed discussion). Too few cases resulted in a decision (4) or submission (8) victory for appropriate statistical analyses to be run, which further emphasises the role of knockout power as the most important avenue to victory in human contest competition.

### Study 2: Discussion

These findings again supported the notion that larger body size in humans is associated with knockout power. Larger individuals are much more likely (200%) to emerge victorious against their lighter counterparts because larger combatants are much more likely (200%) to knock out their lighter counterparts. Because knockouts can result in severe medical problems or death (Flynn et al., 2016; Hånell & Rostami, 2020), this finding is important for: policy-makers who wish to reduce, or continue the reduced occurrence of, fights without weight restrictions; legal researchers, who seek to understand the morphological attributes of perpetrators of single-punch fatalities (Flynn et al., 2016); sports performance researchers, who wish to understand the association between body size and fighting capabilities (Viano et al., 2005; Walilko et al., 2005); medical researchers, who have only recently begun to examine the causes of a knockout (Hånell & Rostami, 2020); and biological anthropologists (Puts, 2010) and evolutionary psychologists (Sell et al., 2009, 2012; Zilioli et al., 2014) interested in the adaptive significance of threat perception based on cues to body size.

For the present work, these findings pave the way for a suite of novel evolutionary psychological predictions. If larger body size was shaped by sexual selection to increase force output in the form of knockout power, then human perceptual systems—if evolved to enable accurate information gathering—should have been shaped by sexual selection to track force output in the form of knockout power from body size. If human dominance judgments predict fighters’ knockout victories, this would suggest that humans can accurately predict the frequency with which fighters have knocked out *similar-sized*—emphasising our conservative approach—adversaries.^2^

## Study 3

### Study 3: Method

Study 3 was conducted as part of a larger, pre-registered project examining the associations between win percentage, facial shape, perceived fighting ability, and perceived aggressiveness (Caton et al., 2022b). All fighters were sourced from the entire available sample on ufc.com, up until April 4, 2020, when the data were collected.

We evaluated the photographs associated with the 715 fighters used in Study 1b. Following our pre-registered protocols, we eliminated fighters whose head was slightly tilted, whose hair had obscured their face, and female fighters. After excluding photographs based on these characteristics, we ended up with a sample of 516 fighters. We then asked MTurk participants to rate these fighters on perceived aggressiveness.

Specifically, we asked 500 US-based MTurkers (*M*_age_ = 38.31, *SD* = 10.79; 297 males, 201 females, two “other”) to rate a random selection of 50 of the 516 facial/body photographs (ufc.com) on their perceived fighting ability (“*How successful is this fighter?*” rated from 1 = *not successful at all* to 7 = *excellent*; intraclass correlation: 0.86). This is often viewed as the more conservative approach in social perception research, in comparison with forced-choice paradigms (Dong et al., 2023; Zilioli et al., 2014). That is, forced-choice paradigms compared to continuous ratings of single photographs have generally been criticised, making it less appropriate to present participants with the openweight fighters of our second study (Dong et al., 2023; Zilioli et al., 2014). MTurk workers held a 95% approval rating for the completion of at least 100 studies (Peer et al., 2014), provided informed consent, and were compensated with $1.00 USD for what was advertised as a 5-minute study. Ethics was approved by the human research ethics committee of the The University of Queensland (project number 2021/HE001045).

Multilevel models were also conducted for the following analyses, with rater identification modelled into the random intercept. Similar results were found (i.e., all analyses in both multiple regression and mediation analyses were significant), but the multilevel models also resulted in a singular fit, and thus the less complex model was preferred. Consequently, multiple regression analyses are reported below.

### Study 3: Results

Human perceptions of fighting ability positively tracked the frequency with which male contestants had knocked out similar-sized adversaries, β = 0.15, *t*(513) = 4.31, *p* < .001, but negatively tracked their submission, β = −0.14, *t*(513) = − 3.86, *p* < .001, and (nonsignificantly) decision, β = −0.04, *t*(513) = − 1.21, *p* = .28) victories, after controlling for total fights. Next, we tested whether the human psychological capacity to perceive men’s knockout power is explained by internal processes that attend to cues of men’s body size.

**Fig. 1:**
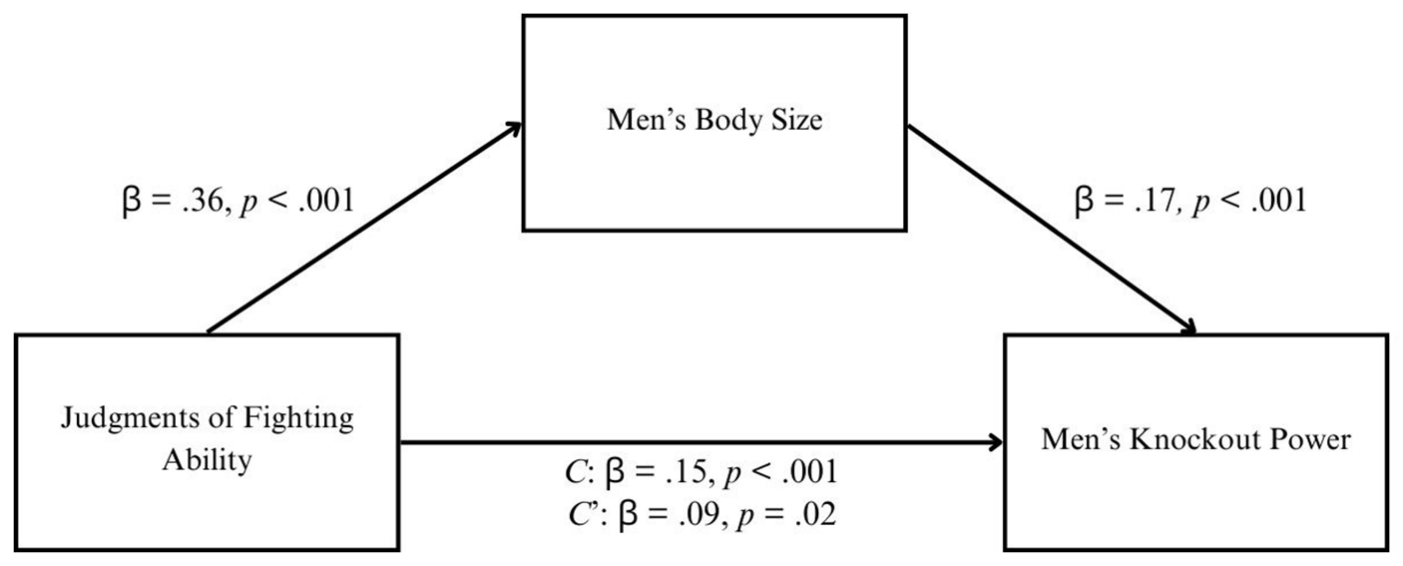
Dominance judgments track men’s knockout power entirely from men’s body size. *Note*: Standardised regression coefficients for the relationship between perceived fighting ability and knockout power as mediated by the fighter’s body size. Total fights was controlled for but is not included in the figure

A mediation analysis (SPSS PROCESS macro; model 4, v.4.1, 10,000 bootstrap samples; Hayes, 2013) was conducted, controlling for total fights. Findings are depicted in Fig. 1. Results of the bias-corrected bootstrapped analyses found that perceived fighting ability had a significant indirect effect on knockout power via body size (*ab* path = 0.06, bootstrap *SE* = 0.02, 95% CI [0.03, 0.10]). The absence of zero within this confidence interval range supports the hypothesis that body size would significantly mediate the association between perceived fighting ability and knockout power. When we accounted for the fighter’s body size, the association between perceived fighting ability and knockout power was reduced (Fig. 1). Because human body size evolved to increase force output, specifically examined here as knockout power for the human evolution of hand-to-hand combat (Carrier & Morgan, 2014; Morgan & Carrier, 2013), human psychology consequently evolved to exclusively track force output (here examined as knockout power) from body size. The full model accounted for 62.8% of the variance in knockout power.

## General Discussion

Since our data demonstrate that human body size may reflect selection for increased force output (which may take the form of knockout power in human hand-to-hand combat), psychological processes for accurately tracking force output in the form of knockout power from body size may also have evolved. Indeed, we found that the association between dominance judgemnts and knockout power was mediated by men’s body size, such that participants were able to track a man’s knockout power by attending to cues of their body size. Our findings provide compelling support for the hypothesis that not only have human morphological features been shaped by sexual selection, but also the psychological systems that enabled accurate information gathering regarding the likely costs and benefits of engaging in conflict (Arnott & Elwood, 2009).

Body mass and size are reliably associated with resource-holding potential across mammals (Arnott & Elwood, 2009). The association we report between body size and knockout power provides compelling evidence that men’s body size is associated with their fighting success (see note 1), upon which much evolutionary psychological research is predicated (Anikin et al., 2021; Aung et al., 2021a; Carrier & Morgan, 2014; Caton et al., 2022a, 2022b; Evans et al., 2006; Fessler et al., 2014; Holbrook & Fessler, 2013; Holzleitner et al., 2014; Kniffin et al., 2019; Puts, 2010; Sell et al., 2017; Thomsen et al., 2011; Zilioli et al., 2014). Only one study of which we are aware investigated a relationship between male body size and knockout power; Baird et al. (2010) found a *negative* association between weight class and knockout frequency. This speaks to a potential limitation in boxing research for the purposes of examining human contest competition since the combination of large padded gloves (Muzzi et al., 2022) and the weaker neck strength of individuals with smaller body size (Catenaccio et al., 2017) may explain the discrepancy between the findings of Baird et al. (2010) and the present work. Further to the present work’s examination of knockout power in MMA contests generally, the pronounced associations found in contests with no weight restrictions—never before examined in the empirical literature—indicate that human body size is a potent measure of resource-holding power in humans.

The present work reports novel psychological associations between knockout power, body size, and dominance judgments that advance animal contest theory more broadly. Animal contest models imply that contestants should possess accurate information about their own and, under certain models, their opponent’s resource-holding power and fighting performance (Arnott & Elwood, 2009). Lane and Briffa (2020) note it is often difficult to measure nonhuman animals’ judgments of their opponent’s resource-holding power, and so there is little information about the accuracy with which animals can judge their opponents. Animal contest research has used motivational probing, wherein an experimenter interrupts the contest and contestants’ latency to re-engage in the fight is measured (see Lane & Briffa, 2020). The present work provides necessary support for the argument that animals—in particular, humans—possess accurate information about their opponent’s resource-holding power.

Human dominance judgments influence a host of social and psychological outcomes—from electoral decisions to criminal sentencing and romantic partner choice (Oosterhof & Todorov, 2008; Todorov et al., 2005; Willis & Todorov, 2006)—and people rate larger men as higher in fighting ability and aggressiveness (Caton et al., 2022b), leadership ability (Holbrook & Fessler, 2013; Re et al., 2012), masculinity (Holzleitner et al., 2014), persuasiveness (Kniffin et al., 2019), and attractiveness (Sell et al., 2017). The present work suggests that these dominance judgments accurately gauge the likelihood with which a potential mate can incapacitate their adversaries, which would have aided in the selection of potential mates, allies, and leaders throughout our species’ evolutionary history. Future research can explore whether perceptions of masculinity (Little et al., 2015), leadership ability (Re et al., 2012), and short- or long-term attractiveness (Little et al., 2015) also predict men’s actual capacity to knock out their rivals. Because male fighters with deep voices have larger bodies (Aung et al., 2021), future research could also explore whether human dominance judgments based on fighters’ formant frequencies track their knockout power.

### Limitations and Future Research Directions

Despite a number of theoretical and practical implications across the biological, psychological, anthropological, and sports sciences, this study is not without its limitations. First, the present work used MMA data, noted as the best form of data currently available to human contest competition research (Caton et al., 2022a, 2022b; Lane & Briffa, 2020; Zilioli et al., 2014). However, MMA combat involves gloves, which mitigates hand injuries and may inflate the importance of knockout punches (Muzzi et al., 2022). MMA research neglects weaponized combat, choking, gouging, lethal strikes (e.g., throat punches, punches to the back of the head), and running during combat across open landscapes. These considerations would have been of great importance over evolutionary time and, importantly, depended on upper body strength (Sell et al., 2012). These considerations underscore the broader argument of the present work that human body size evolved to increase force output in agonistic exchanges, which may take the form of knockout power for hand-to-hand combat specifically.

Second, the nature of contest competition data is such that any fight outcome (e.g., knockout) necessarily precludes other fight outcomes (e.g., submission, decision). This preclusion necessarily occurs in any contest data; if Person A incapacitates Person B via a strike with a hand or a weapon in a hunter-gatherer society, then this precludes other, less immediate forms of victory. Knockouts precluding the winner from winning via other forms of victory emphasise that the benefits associated with increased knockout power may supersede, and therefore preclude, the benefits stemming from other fighting manoeuvres. This does not mean that the human body did not evolve to increase the success of fighting manoeuvres, but that the application of force (in particular for human hand-to-hand combat, via knockout power) can result in immediate death (Flynn et al., 2016). Future research might wish to examine whether increased body size also contributes to increased submission or decision victories in contests where opponents are less likely to be knocked unconscious, such as wrestling. Existing data can be analyzed to examine whether increased body size predicts the type of medal received for male Olympic judo wrestlers in an openweight category, which is beyond the scope of the present work (see RGriffin, 2021, for a dataset of 271,116 Olympic events from 1896 to 2016, including male openweight judo wrestlers, their weight, and medal received). This would demonstrate that increased body size contributes to increased force output in the context of wrestling absent of knockout victories, which would support our broader assertion that male body size evolved to increase the application of force in contests and further support the assertion that knockout power supersedes, and therefore precludes, other forms of victory that are otherwise advantageous in the absence of knocking an opponent unconscious.

Third, the present work found that heavyweight fights indeed end in knockouts more frequently than lighter weight category fights. This is because we found that heavyweight fight divisions exhibit more knockout victories than lighter weight divisions. Given that every fight results in a winner and a loser, the finding that heavyweight fight divisions exhibit more knockout victories necessarily means that the heavyweight fights exhibit more knockout losses than lighter weight divisions. This does not mean that these data violate independence of observation assumptions (see Caton et al., 2022a, which addresses this issue), and Study 2 addresses this consideration empirically using individual fight data (as in Caton et al., 2022a). While beyond the scope of the present work, a potential moderator of this analysis is knockout resistance (and for submissions, submission resistance) which has only been given theoretical consideration (e.g., Carrier & Morgan, 2014) and remains to be empirically examined. Future research may wish to use geometric morphometric analyses to examine the association between facial (Caton et al., 2022b) or neck (Caton & Lewis, 2021) morphology on the association between body size and knockout resistance, demonstrating how this association varies as a function of other morphological features.

Fourth, human contest competition research via competitive sports data has only recently emerged, and the present work focussed on body *size*—the allometric system responsible for an organism’s *overall* development—as one of the most commonly used measures in animal contest research (Arnott & Elwood, 2009) and the overarching cue to which our psychological systems attend (Sell et al., 2009, 2012, 2017). Nonhuman animal research has also explored the role of body mass (e.g., represented as body mass index in humans, in which weight is divided by height in metres squared; see Arnott & Elwood, 2009, which lists nonhuman contest research according to whether the study examined body size [i.e., larger animals] or body mass [i.e., heavier animals]) and weapon size (Arnott & Elwood, 2009) whereas laboratory studies in humans have also examined the associations between upper-body strength and social and psychological outcomes (Hill et al., 2013; Kordsmeyer et al., 2018; Sell et al., 2009, 2016).

Fifth, we note that human bodies represent complex phenotypes, wherein large numbers of correlated morphological traits combine to determine the strength of sexual selection (Caton & Dixson, 2022b; Caton et al., 2022a). Indeed, a shortcoming of the literature examining how sexual selection has shaped morphological features—particularly facial-morphology-involved agonistic displays and the suggested underlying formidability—have relied of a small number of measures or ratios to characterize physique (Caton & Dixson, 2022b; Caton et al., 2022a). Ultimately, the present work examined body size, psychological perceptions, and knockout frequency, which would have been otherwise ethically unfeasible in most settings other than with publicly available data, which do not contain strength-based measurements.

## Conclusion

Human body size represents a reliable cue of increased knockout power, and human psychology consequently evolved to track force output, which may take the form of knockout power, from body size. Humans can track the frequency with which men have knocked out their rivals through attending to cues of men’s body size. Human dominance judgments—important to numerous psychological domains, including attractiveness, leadership, and legal decision-making (Oosterhof & Todorov, 2008; Todorov et al., 2005; Willis & Todorov, 2006)—accurately predict the likelihood with which a potential mate, ally, or rival can incapacitate their adversaries.

### Electronic Supplementary Material

Below is the link to the electronic supplementary material.

Supplementary Material 1

## Data Availability

All data used are available from the corresponding author upon request.

## Footnotes

1 Previous research on body size and fighting success has been limited. Using self-report data, Felson (1996) asked participants to describe a physical encounter from their past and found that men who report being stronger and larger than their opponent were four and a half times more likely to also report injuring their opponent. Evolutionary biology research has often focussed on biomechanics paradigms (e.g., Viano et al., 2005; Walilko et al., 2005) or, when using or competitive sports paradigms, has been restricted by methodological problems (e.g., Aung et al., 2021; Palmer-Hague et al., 2018). Laboratory paradigms are difficult to generalize to an organism’s resource-holding power because these methodologies do not account for the counterargument that competing organisms might possess morphological structures (e.g., strong craniofacial morphology; Carrier & Morgan, 2014) or behavioral systems (e.g., fighting skill; Lane & Briffa, 2020) shaped by intrasexual selection to reduce or overcome the competing organism’s body size advantage. Competitive contest data accounts for this counterargument, and mixed-martial-arts data has commonly used in evolutionary psychological (Aung et al., 2021a; Caton et al., 2022b; Zilioli et al., 2014) and biological (Lane & Briffa, 2020) research as a proxy for the environment in which humans evolved (Zilioli et al., 2014). Second, Aung et al. (2021a, 2021b) found that size (which was a latent variable of both height and body size) predicted fighting success (which was a latent variable of win percentage, retirement status, and Elo ratings). Palmer-Hague et al. (2018) found that BMI was positively associated with number of fights and number of wins, but not proportion of wins. Both results must be interpreted in light of fighters being positioned within weight classes: these past findings confusingly suggest that heavyweight fighters will win more often against heavyweight fighters than lightweight fighters will win against lightweight fighters. We contend that this makes overall wins an inappropriate measure of resource-holding power in weight-restricted data when examining the association between body size and resource-holding power. Knockout wins would serve as a stronger *alternative* measure of resource-holding power in weight-restricted data. If men’s body size is positively associated with their knockout victories in weight-restricted data, where there is minimal (if any) variation in body size within the weight class, then this would indicate that heavier fighters are more able than lighter fighters to knock out similar-sized opponents. Knockout victories serve as a more appropriate measure of resource-holding power in weight-restricted data. Third, we searched Google Scholar for current research on body size and knockout power specifically. We searched for the terms “knockout power” or “knockout wins” combined with “body size,” “weight class,” “pounds,” or “kilograms,” and also the related but distinct “body mass” or “body mass index.” To our knowledge, there are no studies that have discovered a positive association; only one study of which we are aware investigated a relationship between male body size and knockout power, in which Baird et al. (2010) found a *negative* association between weight class and knockout frequency. Minimal research on this topic is to be expected, given the ethical problems of conducting such a study and the recency of publicly available data.

2 Because Study 3 contains weight-restricted data, these findings must be interpreted such that humans perceive the *frequency* (i.e., knockout wins, controlling for total fights) with which fighters have knocked out similar-sized adversaries. If we had instead presented participants with the fighters in Study 2, then participants’ selection of the fighter who successfully knocked out their opponent could instead be due to the statistical chance associated with forced-choice paradigms: by default, participants have a 50% chance of selecting the successful fighter. This is not the case in paradigms which require participants to rate photographs on a continuous scale, one at a time.
