# Supplementary material for: Human Male Body Size Predicts Increased Knockout Power, Which Is Accurately Tracked by Conspecific Judgments of Male Dominance
Source: Hum Nat. 2024 Jun 15;35(2):114–33. doi: 10.1007/s12110-024-09473-7 (PMC11317448; doi:10.1007/s12110-024-09473-7)
Supplement: Supplementary file 1 — Supplementary Material 1 [file 12110_2024_9473_MOESM1_ESM.docx]

Electronic Supplemental Material (ESM)

for

Human Male Body Size Predicts Increased Knockout Power, Which Is Accurately Tracked by Conspecific Judgments of Male Dominance

*Human Nature* 35(2), 2024. https://doi.org/10.1007/s12110-024-09473-7

Neil R. Caton^1,*^, Lachlan M. Brown^1^, Amy A. Z. Zhao^1^, Barnaby J. W. Dixson^2^

^1^ School of Psychology, University of Queensland, Brisbane, Queensland, Australia.

^2^ School of Psychology, The University of the Sunshine Coast, Sunshine Coast, Queensland, Australia.

*Correspondence to [n.caton@uqconnect.edu.au](mailto:n.caton@uqconnect.edu.au)

**Study 1: Supplemental Results**

**Study 1a**

Controlling for total fights, submission victories, and decision victories, there was a significant positive association between body size and knockout victories, β = .05, *t*(5114) = 6.71, *p* < .001. Controlling for total fights, knockout victories, and decision victories, there was a significant negative association between body size and submission victories, β = −.03, *t*(5114) = −4.93, *p* < .001. Controlling for total fights, knockout victories, and submission victories, there was a significant negative association between body size and decision victories, β = −.11, *t*(5114) = −12.12, *p* < .001. There was no evidence of multicollinearity in any of these models as indicated by VIF scores below 10 (Hair et al., 1995).

**Study 1b**

Controlling for total fights, submission victories, and decision victories, there was a significant positive association between body size and knockout victories, β = .08, *t*(710) = 4.77, *p* < .001. Controlling for total fights, knockout victories, and decision victories, there was a non-significant association between body size and submission victories, β = .03, *t*(710) = 1.53, *p* = .13. Controlling for total fights, knockout victories, and submission victories, there was a significant negative association between body size and decision victories, β = −.05, *t*(710) = −2.44, *p* = .02. There was no evidence of multicollinearity in any of these models as indicated by VIF scores below 10 (Hair et al., 1995).

**Study 2: Results**

**Knockout Power**

There were too few cases of female fights that ended in a knockout (1) to conduct an interaction. It could be argued that, in this specific instance of a singular fight which prevents conducting an interaction, it might be more appropriate to run the analysis without the singular female fight; it would not be akin to following up simple effects analyses in the presence of a non-significant interaction because no interaction could be conducted. But even after excluding this singular female fight, there was still a significant association between the focal fighter’s body size on the likelihood of the focal fighter knocking out their opponent, controlling for the non-focal fighter’s body size (*OR* = 1.06, *Z* = 2.12, *p* = .03).

**Additional Combat Metrics**

Here, we addressed whether there existed any other additional combat advantages to increased body size rather than raw force output. Focal fighters who were heavier (vs. lighter) than their opponent were no more likely to attempt strikes (β = −.23, *t*(42) = −1.53, *p* = .13; controlling for the non-focal fighter’s attempted strikes: β = .06, *t*(41) = .46, *p* = .65), land strikes (β = −.13, *t*(42) = −.87, *p* = .39; controlling for the non-focal fighter’s landed strikes: β = −.02, *t*(41) = −.09, *p* = .93), attempt takedowns (β = .16, *t*(42) = −1.03, *p* = .31; controlling for the non-focal fighter’s attempted takedowns: β = −.09, *t*(41) = −.54, *p* = .59), or land takedowns (β = .07, *t*(42) = −.44, *p* = .67; controlling for the non-focal fighter’s landed takedowns: β = .04, *t*(41) = −.26, *p* = .80). Results did not change when we used focal fighter’s body size (kilograms) as our continuous predictor of interest, controlling for the non-focal fighter’s body size (attempted strikes: β = .12, *t*(41) = −.62, *p* = .54; controlling for the non-focal fighter’s attempted strikes: β = .18, *t*(40) = 1.22, *p* = .23; landed strikes: β = .10, *t*(41) = .49, *p* = .63; controlling for the non-focal fighter’s landed strikes: β = .23, *t*(40) = 1.15, *p* = .26; attempted takedowns: β = −.12, *t*(41) = −.59, *p* = .56; controlling for the non-focal fighter’s attempted takedowns: β = −.03, *t*(40) = −.14, *p* = .89; landed takedown: β = .004, *t*(41) = .02, *p* = .98; controlling for the non-focal fighter’s landed takedowns: β = −.03, *t*(41) = −.14, *p* = .89). Heavier combatants possess greater RHP through their capacity to generate knockout power.

**Reference**

Hair, J. F. Jr., Anderson, R. E., Tatham, R. L. & Black, W. C. (1995). *Multivariate data analysis* (3rd ed). New York: Macmillan.
